# Leveraging a national cloud-based intensive care registry for COVID-19 surveillance, research and case-mix evaluation in Brazil

**DOI:** 10.5935/0103-507X.20220016-en

**Published:** 2022

**Authors:** Amanda Quintairos, Ederlon Alves de Carvalho Rezende, Marcio Soares, Suzana Margareth Ajeje Lobo, Jorge Ibrain Figueira Salluh

**Affiliations:** 1 Department of Critical Care and Postgraduate Program in Translational Medicine, Instituto D’Or de Pesquisa e Ensino - Rio de Janeiro (RJ), Brazil.; 2 Department of Critical Care, Hospital do Servidor Público Estadual de São Paulo -São Paulo (SP), Brazil.; 3 Department of Critical Care, Hospital de Base de São José do Rio Preto, Faculdade de Medicina de Rio Preto - São José do Rio Preto (SP), Brazil.

## INTRODUCTION

Large national databases of intensive care units (ICUs) generate valuable information for the management and guidance of public policies.^(1)^ These national ICU registries were established in high-income countries more than 20 years ago. Their contribution to the understanding of case mix and outcomes of ICU patients as well as to clinical research and quality improvement in these countries is substantial.^(1)^ More recently, initiatives have successfully implemented national registries in Latin America^(2)^ and Asia.^(3)^

Brazil is the largest country in Latin America, with an estimated population of 213,209,356. In 2015, the *Associação de Medicina Intensiva Brasileira* (AMIB) established the “Brazilian ICUs Project” (www.utisbrasileiras.com), leveraging from the existing national database of case mix and quality assurance for ICUs. ^(4,5)^ Participation is voluntary and grants access to a free version of the software. It uses cloud-based software to continuously track quality and performance indicators.^(6)^ The database now includes information on more than 4,000,000 adult ICU admissions.

In the present article, we describe the role of “*UTIs Brasileiras*”, a project that currently involves 469 hospitals, 1,154 adult ICUs and 22,832 ICU beds (31.6% of total ICU beds in Brazil). We discuss the Brazilian intensive care response in the coronavirus disease (COVID-19) pandemic, highlighting aspects of research and evaluating case mix and outcomes as well as trends in patterns of care and ICU resource utilization. Although the database represents a large number of ICUs and ICU beds and a significant share of the country’s critically ill patients, it does not reflect (in the epidemiologic sense) a perfect picture of the country. We acknowledge that a concentration of ICUs in the database are from the southeast region (although this is where most Brazilian ICUs are located).^(7)^

### Multicentric research

A fast research response during the pandemic required centralized access to data, customization of new datasets, and obtaining ethics approval and funding (Table 1). The Brazilian intensive care community responded by conducting clinical trials^(8-10)^ and observational studies. The national ICU registry, through its shared dataset, incorporated COVID-19-related variables, and subsequently, performed studies were designed to evaluate case mix and outcomes,^(5)^ trends in the use of resources,^(5)^ the validity of current scoring systems^(11)^ and the impact on non-COVID ICU patients.^(12)^

**Table 1:** The response to the pandemic and future challenges of the Brazilian intensive care unit registry

	Prepandemic status	Response	Limitations and future challenges
Research	Allowed local and multicenter studies through a shared electronic database	Fast coordination among centers for observational research Incorporation of COVID-19-related variables in dataset	Need for local IRB approval at each center, limiting wider inclusion Registry-based trials (integration with randomized studies)
Case-mix and resource use evaluation	Local and national perspective	Creation of COVID-19 analytics and reports	Integration with public health databases
Surveillance	Allowed local surveillance of resource use and bed availability	Creation of COVID-19 dashboards of cases, ICU beds and resource use	Integration with total caseload Georeferencing
Outcomes	Detailed in-hospital outcomes for general patients and main diagnostic categories	Availability of ICU and hospital outcomes of COVID-19 patients, including subsets (ventilated, nonventilated, dialysis)	Need for long term (posthospital vital status) Incorporating patient-centered outcome measures (quality of life, functionality, cognitive status)

IRB - Institutional Review Board; ICU - intensive care unit.

Kurtz et al. analyzed data from 126 ICUs and showed changes in patient characteristics during the first 8 months of the pandemic.^(5)^ Age and mortality rates declined as noninvasive respiratory support (noninvasive ventilation - NIV and high flow nasal cannula - HFNC) was increasingly used and adjusted 60-day hospital mortality decreased. This provided observational evidence of the safety of NIV as a ventilatory strategy for select patients.

Regarding outcomes, several scoring systems have been evaluated and developed for COVID-19.^(13)^ In Brazil, the Simplified Acute Physiology 3 (SAPS 3) score is used as a standard in ICUs, and it allows comparisons between the prepandemic and pandemic risk-adjusted outcomes. Sudden increases in the standardized mortality rates (SMRs) were observed and were initially ascribed to excess deaths. Brazilian data of 30,571 patients in 126 ICUs^(14)^ demonstrated that increases in SMRs occurred in part because traditional scoring systems are not well calibrated for COVID-19 patients. However, disruption in the organization of delivery of critical care has worsened overall ICU performance.^(11,12)^ This led to the hypothesis that excess deaths could also be present among critically ill non-COVID patients. A study using data from the registries of 42 hospitals included 514,219 ICU admissions from 2011 - 2020. In this study, hospital outcomes of critically ill non-COVID-19 patients worsened during the pandemic in 2020, after nine years of a downward trend, resulting in an increased number of deaths in critically ill non-COVID patients even after risk adjustment, especially during surges of COVID-19.^(12)^

### Case mix, resource use and outcomes of COVID-19 patients admitted to the intensive care unit during the pandemic in Brazil

Eighteen months since the pandemic began, there were more than 20,000,000 people infected with the virus and more than 594,000 deaths in Brazil. Therefore, real-world ICU data were needed to understand the burden of COVID-19 and its clinical patterns to support preparedness and planning. The Brazilian ICU Registry compiled data from 192,500 ICU admissions of COVID-19 patients over an 18-month period (Table 1S - Supplementary material). Initially, patients were older, ranging from 60.5 years in the first quarter of the pandemic to 54.7 years recently. The mean hospital mortality ranged from 34.2%, with a peak of 43.9% from March to May 2021, decreasing again to 35.3% (Table 1S - Supplementary material). During the second wave (1st quarter of 2021), we observed the highest number of ICU admissions, probably associated with the increased circulation of the P1/Gamma variant of SARS-CoV-2, among other factors. In this period, the use of invasive mechanical ventilation (MV) increased to include 57.7% of ICU patients. The in-hospital mortality of MV patients remained high during the eighteen months (59.8% to 68.2%). Major increases in mortality and the use of MV were observed during the months when there were a higher number of cases in ICUs^(15)^ and in the community, probably reflecting the saturation and collapse of the health care system, especially in public hospitals (Table 2S - Supplementary material).

In the Brazilian ICU Registry, we also observed a significant increase in the use of NIV over time (Table 1S - Supplementary material), reflecting the incorporation of new evidence^(5)^ generated throughout the pandemic, which allowed better allocation of ICU resources.^(16)^ The Brazilian ICU Registry also reflected the trends in the use of renal replacement therapy and MV (during the peaks of the pandemic as well as compared to data from nonCOVID-19 patients from previous years). This information was readily available for ICUs and made public through the registry’s website (www.utisbrasileiras.com), which was updated weekly. Understanding such local, regional and national trends potentially facilitated the better management of resources (sedatives, vasopressors, renal replacement therapy supplies, ventilators and human resources, for example) and preparation of the ICUs.

As we already mentioned, despite intrinsic limitations in performance in COVID ICUs, the worsening of performance in general ICUs is evident. Additionally, we observed differences in nonadjusted outcomes such as mortality in nonventilated (7.6% *versus* 17.9%) and ventilated (60.5% *versus* 73.2%) patients, overall ICU mortality (29.5% *versus* 51.9%) and hospital mortality (30.8% *versus* 53.9%). The Brazilian national registry made noticeable the need to strengthen the public system and its processes in the delivery of care to critically ill patients. Furthermore, detailed information on resource use and outcomes in Brazilian public and private ICUs is provided in table 2S of the supplementary material. However, more studies are required on this topic, as the nature of the database may have biased the selection of ICUs, and the interpretation of the findings merit an adjusted analysis.

Longitudinal data showed changes in case mix, resource use and patterns of care. In Brazil, among the 192,500 COVID-19 ICU admissions, the mean hospital LOS was 13.4 days at the beginning of the pandemic, decreasing to 11.8 days in the period from June to August 2021 (Table 1S - Supplementary material). It is important to note that gradually, younger patients with fewer comorbidities and with lower severity of illness were admitted to ICUs more frequently in the beginning of 2021. Similarly, frailty was initially seen in 13.5% of patients admitted to the ICU from March to May 2020 and decreased to 7.5%. This finding is possibly explained by the efficacy of a national vaccination program for elderly individuals (vaccination started with elderly patients in January 2021 and was available for all those older than 30 years by June 2021).

**Figure 1:**
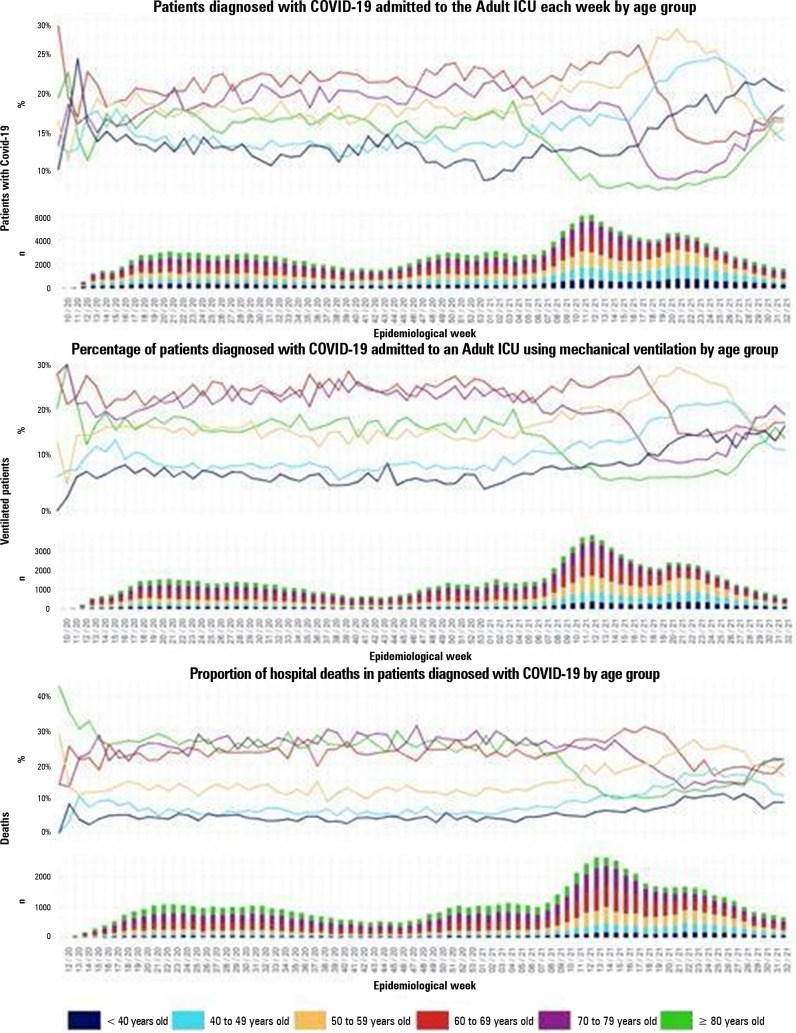
Trends among COVID-19 adult intensive care unit patients by resource use and mortality per age group. ICU - intensive care unit.

Figure 1 shows the trends of COVID-19 patients admitted to adult ICUs by resource use and mortality per age group. This shows a significant reduction in elderly patients, as well as their resource use and mortality, following the vaccination period. However, in the last quarter, it also started to show a small, albeit consistent, rebound effect in the age groups sequentially prioritized in the national vaccination program. This has motivated investigation on the waning effects of vaccines over time due to immunosenescence as well as a discussion and implementation of booster doses for these populations. Even though dates and strategies of the national vaccination program are public information, the registry does not have specific data on vaccinated patients, and this can be considered a limitation of this study.

Data also provided insights into ICU performance during the pandemic. We observed a significant shift in the SMR compared to previous years (0.97 in 2019 to 1.24 in 2020 and 1.54 in 2021), demonstrating worsethan-expected ICU performance.^(17)^ In addition to all the challenges associated with the elevated number of severe cases of a previously “unknown” condition, national health policies may have contributed to the observed results. The lack of central government leadership and coordination has resulted in the failure to implement more effective strategies to suppress the spread of the virus, leading to an initial surge that progressed into sustained community transmission that overwhelmed hospitals and ICUs for several months, resulting in mortality that remained high throughout the analyzed period. This was a major challenge to the resilience of the entire health system, including intensive care.

### The postpandemic perspective

Several lessons were learned during the pandemic, and they impose new challenges for our future. Increasing the reach of an ICU registry in a large country is important, and finding ways to integrate a larger number of ICUs in research should be a goal (Table 1). It would not only allow a more accurate case-mix evaluation and more patients for research but also provide a sustainable surveillance platform for ICUs. In addition, linkage with other public health databases would allow follow-up of posthospital outcomes as well as the use of new outcome measures that incorporate quality of life and functional status among other relevant patient and family-centered outcomes.

## CONCLUSION

The availability of structured clinical data and near real-time information from the registry enabled intensive care units and intensive care unit networks to manage bed occupancy and resource availability. It also served as a tool to export local intensive care unit data for clinical research demonstrating outcomes as well as improving the understanding of performance analysis during the pandemic. In addition, its existence allowed a countrywide perspective on the epidemiology and progression of COVID-19 cases requiring intensive care unit admission using real-world data to inform on changes in case mix, trends in treatments and resource use in intensive care units.
