# Supplementary material for: Leveraging a national cloud-based intensive care registry for COVID-19 surveillance, research and case-mix evaluation in Brazil
Source: Rev Bras Ter Intensiva. 2022 Apr-Jun;34(2):205–9. doi: 10.5935/0103-507X.20220016-en (PMC9354116; doi:10.5935/0103-507X.20220016-en)
Supplement: Supplementary file 1 [file rbti-34-02-0205-suppl01.pdf]

Leveraging a national cloud-based intensive care registry for COVID-19 surveillance, research and case-mix evaluation in Brazil

Utilização de um registro nacional de terapia intensiva baseado em nuvem para vigilância, pesquisa e avaliação do perfil dos casos de COVID-19 no Brasil

Amanda Quintairos[1], Ederlon Alves de Carvalho Rezende[2], Marcio Soares[1], Suzana Margareth Ajeje Lobo[3], Jorge Ibrain Figueira Salluh[1]

Table 1S - COVID-19 patients clinical demographic characteristics, severity and resource use in Brazilian intensive care units

	Mar - May 2020	Jun - Aug 2020	Sep - Nov 2020	Dec 2020 - Feb 2021	Mar - May 2021	Jun - Aug 2021
Clinical demographic characteristics						
Admissions	18,750	29,728	21,015	35,415	61,630	25,962
Sex						
Male*	60.1	58.8	59.6	58.8	59.0	61.1
Female*	39.9	41.2	40.4	41.2	41.0	38.9
Age (years)†	60.5	61.4	61.5	61.9	57.5	57.4
31 - 40*	10.9	9.2	9.1	8.7	11.5	16.1
41 - 50*	15.2	13.8	13.5	14.0	18.5	22.6
51 - 60*	19.3	18.4	18.5	18.5	23.4	22.3
61 - 70*	20.0	22.4	22.8	22.9	22.1	13.2
71 - 80*	16.7	18.5	19.0	19.2	13.9	11.1
81 - 90*	11.1	11.2	10.9	11.2	5.8	7.0
> 90*	3.0	2.6	2.4	2.4	1.2	1.9
Comorbidities	74.0	75.7	76.8	75.3	70.2	63.7
Modified Frailty Index (MFI ≥ 3)*	13.5	14.3	13.4	12.0	7.5	7.5
SAPS 3†	49.3	49.9	48.4	49.0	48.6	46.2
SOFA†	3.2	2.9	2.5	2.7	3.4	2.8
Resource use						
NIV*	18.7	27.4	37.1	39.0	45.7	47.1
MV*	51.2	48.4	41.7	48.1	57.7	48
MV (days)†	14.3	14.8	15.8	14.4	13.2	12.2
Vasopressor*	38.8	33.9	29.2	33.5	38.7	30.8
RRT*	17.8	13.9	8.9	10.4	12.0	9.2
Outcomes						
ICU LOS (days)†	13.4	13.5	13.4	12.8	13.1	11.8
Hospital LOS (days)†	27.3	25.8	23.8	22.6	21.8	17.0
ICU mortality*	32.2	34.6	30.6	36.4	42.7	34.8
Hospital mortality (all patients)*	34.2	36.5	32.2	38.1	43.9	35.3
No ventilated patients' mortality*	7.5	9.9	8.3	10.1	11.8	8.7
Ventilated patients' mortality*	59.8	65.1	65.4	68.2	67.1	65.3
SMR, SAPS 3	1.59	1.64	1.58	1.80	2.09	2.02

SAPS3 - Simplified Acute Physiology Score 3; SOFA - Sequential Organ Failure Assessment Score; NIV - non-invasive ventilation; MV - mechanical ventilation; RRT - renal replacement therapy; ICU - intensive care unit; LOS - length of stay; SMR - standardized mortality ratio. * %; †Median.

Table 2S - COVID-19 patients resource use and outcomes in Brazilian public and private intensive care units

	All	Private	Public
Hospital discharges	186,613	125,847	60,766
NIV*	38.4	39.9	35.3
MV*	50.7	44.0	64.4
MV (days)†	14.0	15.1	12,3
Vasopressor*	34.8	29.8	45.1
RRT*	11.9	10.2	15.3
ICU LOS (days)†	13.0	13.0	13.0
Hospital LOS (days)†	22.9	22.1	24.6
ICU mortality*	36.7	29.5	51.9
Hospital mortality (all patients)*	38.2	30.8	53.9
No ventilated patients' mortality*	9.9	7.6	17.9
Ventilated patients' mortality*	65.7	60.5	73.2
SMR, SAPS 3	1.84	1.57	2.31

NIV - non-invasive ventilation; MV - mechanical ventilation; RRT - renal replacement therapy; ICU - intensive care unit; LOS - length of stay; SMR - standardized mortality ratio; SAPS3 - Simplified Acute Physiology Score 3. * %; † Median.
